# Health, Economy, and Environment: Sustainable Energy Choices for a Nation

**DOI:** 10.1289/ehp.11602

**Published:** 2008-06

**Authors:** Julia M. Gohlke, Sharon H. Hrynkow, Christopher J. Portier

**Affiliations:** National Institute of Environmental Health Sciences, National Institutes of Health, Department of Health and Human Services, Research Triangle Park, North Carolina, E-mail: portier@niehs.nih.gov

Energy policies are in transition worldwide based on a convergence of factors including static oil production coupled with increased demand, a desire for energy independence, and growing awareness of climate change. Making energy choices that improve human health, the environment, and economic development is possible if we understand the complex interplay between systems for energy delivery and sustainable, healthy human environments.

Reducing energy consumption should be the first step. According to the International Energy Agency (IEA 2006), the average American consumes about 7,800 kg of oil equivalent energy per year compared with Switzerland, where the average person consumes 3,700 kg. If we simply apply lessons learned in Switzerland to our energy use, we could conceivably cut consumption in half without altering the quality of life. This drop in consumption will reduce the incidence of a number of diseases affected by energy production (Figure 1).

Energy alternatives lead to surprisingly complex analyses regarding efficiency. For example, some studies have found that substituting biofuels for gasoline will reduce greenhouse gases because biofuel feedstocks sequester carbon during growth. However, other analyses have found that by including land-use change in the analyses, biofuel production could result in a net doubling of greenhouse gases over the next 30 years (Fargione et al. 2008; Searchinger et al. 2008). Moreover, certain biofuels will have a significant impact on water use patterns, food crop production, and deforestation, all of which can have direct and indirect impacts on human health (Figure 1). In addition, biofuels will produce a new mix of air pollutants that have not been extensively studied and could lead to increased air pollution related mortality (Jacobson 2007).

Even seemingly clean sources of energy can have implications on human health. Wind energy will undoubtedly create noise, which increases stress, which in turn increases the risk of cardiovascular disease and cancer. The manufacturing process for photovoltaic panels to produce solar energy results in occupational exposures to silica dust or cadium (Fthenakis et al. 2008). Increased reliance on nuclear fission carries known radiation risks during the generation of electricity and disposal of used fuel. Even hydroelectric energy affects human and environmental health, as noted in several recent articles about the Three Gorges Dam (e.g., Hwang et al. 2007).

We must combine the lessons we have learned in systems approaches in biology, ecology, engineering, and economics to develop a new systems theory, one that, when properly implemented, can begin to identify how changes in our energy policies will impact the health of our nation. To this end, we provide a simple example exemplifying the utility of comparing global health impacts across energy sources (Table 1). Table 1 shows that the use of traditional biomass accounts for greater mortality than other energy sources. Current world consumption of oil has the greatest impact on climate change, whereas predictions suggest that coal-fired power plants account for > 90% of mortality associated with electricity generation.

However, we cannot draw robust conclusions from current analyses of available quantitated health impacts of energy systems because they do not incorporate important factors known to mediate health. For example, health end points related to food shortages resulting from unsustainable biofuel production have not been measured. Links between mercury found in most coal stocks and a range of health end points have not been fully addressed, and combustion products of alternative fuels, including biodiesel and ethanol, as related to health are not fully understood. Occupational exposures in the development and distribution of new fuels is another area of priority and one that calls for expanded focus. Therefore, there is a critical need for a large-scale collaborative effort between social, environmental, physical, engineering, and human health scientists to evaluate risks and benefits associated with rapidly changing energy policies.

The National Resource Council (2007) recently suggested that “an appropriate institutional structure that fosters multidisciplinary intramural and extramural research is needed” to take full advantage of the “revolutions in biology and biotechnology.” Their vision focused on systems biology and laboratory investigation. Expanding this vision to address broad environmental linkages to health will result in fuller descriptions of the health implications due to the social, ecological, and economic changes linked to our changing global environment.

## Figures and Tables

**Figure 1:**
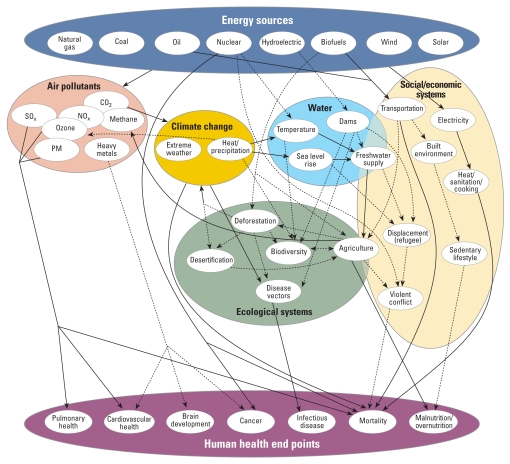
Direct and indirect routes by which energy sources may affect human health. Abbreviations: CO_2_, carbon dioxide; NO_x_, nitrogen oxide; PM, particulate matter; SO_x_, sulfur oxide. Solid lines indicate health impacts that have been quantitated, and dashed lines indicate qualitative evidence.

**Figure:**
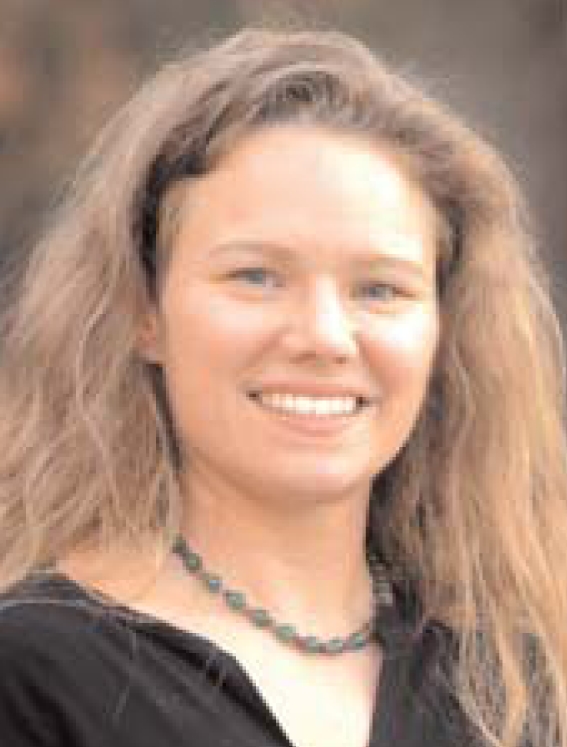
Julia M. Gohlke

**Figure:**
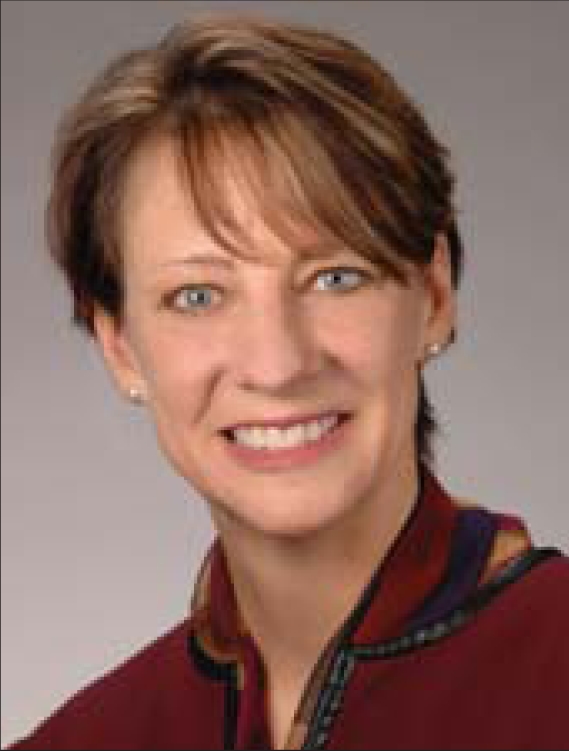
Sharon H. Hrynkow

**Figure:**
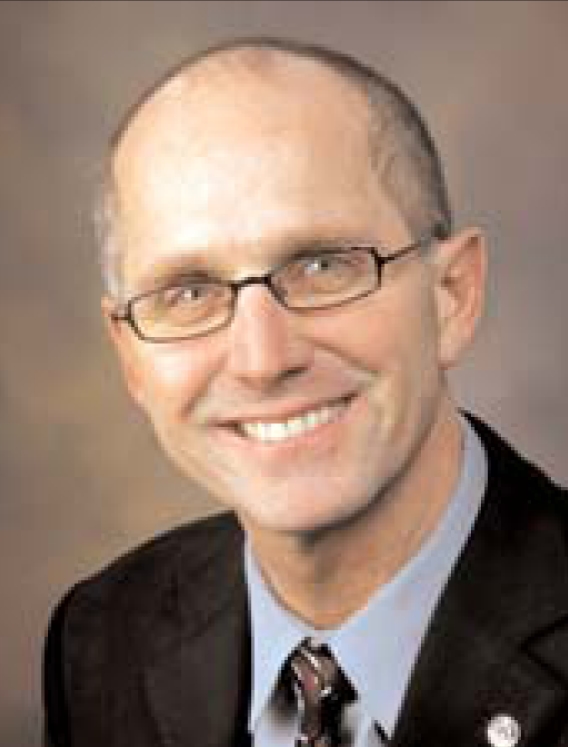
Christopher J. Portier

**Table 1:** A comparison of mortality attributable to energy sources using point estimates from available models.

Energy source	Proportion of world energy use (%)^a^	Full energy chain CO_2_ equivalent (g/kWh)^b^	Climate change–related deaths in 2000^c^,^d^	Electricity generation–related mortality (deaths/TWh)^e^	Power generation–related mortality (deaths/year)	Mortality attributed to transport-related outdoor air pollution (deaths/year)^c^	Total deaths/year
Oil	35.1	1,300	46,340	18.43	8,846.4	651,706	706,892
Coal	22.6	1,000	22,952	24.62	195,778.24	—	218,730
Natural gas	21.7	1,250	27,547	2.821	8,440.432	—	35,987
Nuclear	6.9	20	140	0.074	228.512	—	369
Hydroelectric	2.3	350	818	—	—	—	818
Traditional biomass	9.3	100	944	—	1,497,000^c^	—	1,497,000
Modern biomass	1.4	100	142	4.63	0	—	142
Wind	4.0	75	305	—	—	—	305
Solar	4.0	200	812	—	—	—	812

Abbreviations: kWh, kilowatt-hours; TWh, terawatt-hours.

a Data from Goldemburg et al. (2005).

b Data from the IAEA (2001).

c Data from Ezzatti et al. (2004).

d Assuming that two-thirds of climate change is due to energy source emissions (Intergovernmental Panel on Climate Change 2007).

e Data from the European Commission (2005), assuming that ExternE evaluation is applicable globally.
